# Supplementary material for: The Construction and Exploration of a Comprehensive MicroRNA Centered Regulatory Network in Foxtail Millet (Setaria italica L.)
Source: Front Plant Sci. 2022 May 6;13:848474. doi: 10.3389/fpls.2022.848474 (PMC9121102; doi:10.3389/fpls.2022.848474)
Supplement: Supplementary file 7 [file Data_Sheet_7.zip › Sit-miRN3027_Seita.3G247200.1_795_TPlot.pdf]

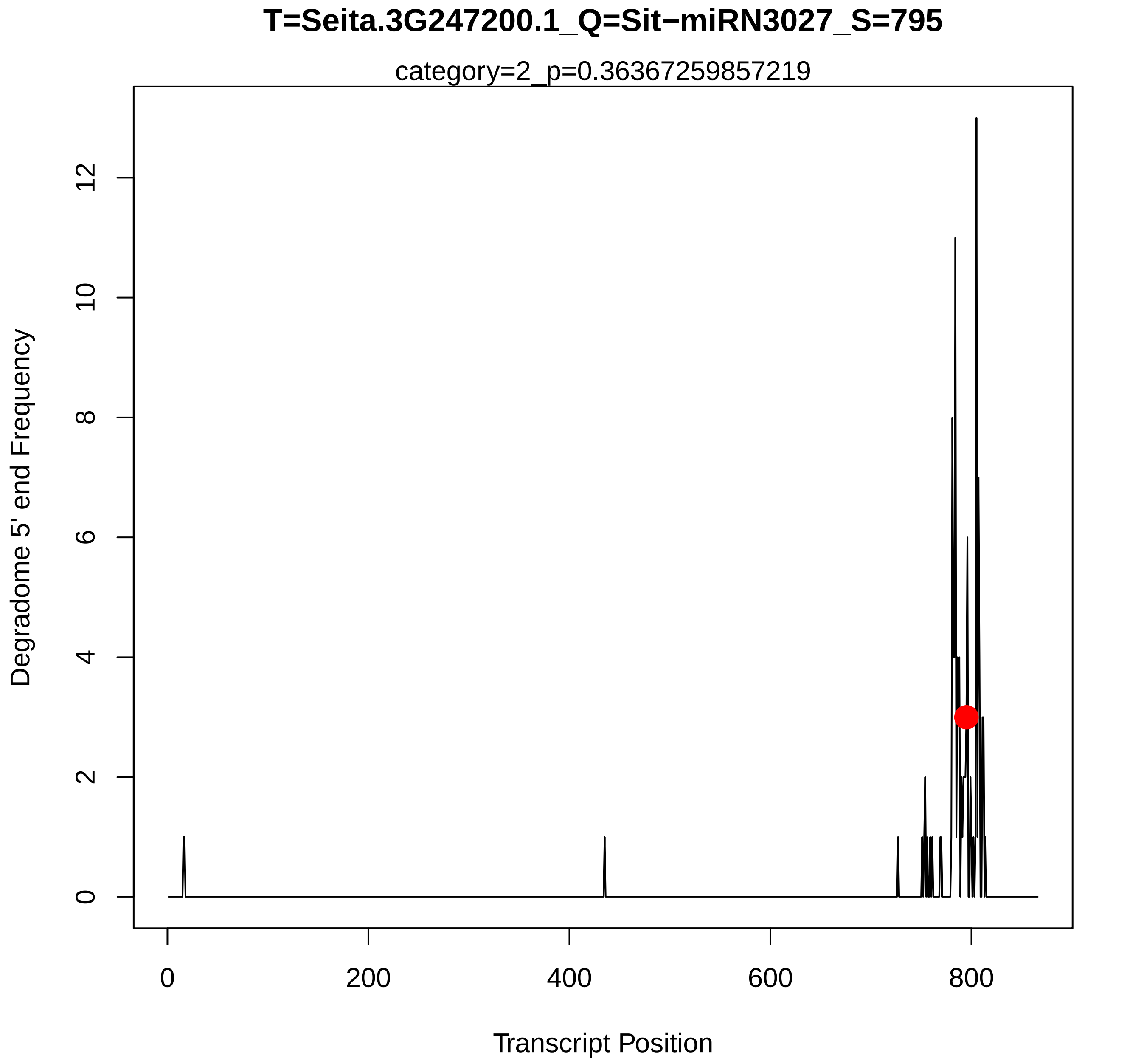

T=Seita.3G247200.1_Q=Sit−miRN3027_S=795
category=2_p=0.36367259857219
Degradome 5' end Frequency
0
2
4
6
8
10
12
0
200
400
600
800
Transcript Position
